# Product News

**DOI:** 10.1155/S1463924679000341

**Published:** 1979


**3rd European Congress of Clinical Chemistry**
June 4-8, Brighton, U.K.
*Dr. P. J. N. Howarth, Department of Chemical Pathology, Guy's Hospital, Medical School, London SE1 9RT, U.K.*

**2nd World Chromatography Conference**
June 5-6, Lisbon, Portugal.
*V. M. Bhatnagar, P.O. Box 1779, Cornwall, Ontario K6H 5V7, Canada.*

**10th International Symposium on Chromatography and Electrophoresis**
June 19-20, Venice, Italy.
*Dr. Alberto Frigerio, Instituto di Riccerche Farmacologiche "Mario Negri," Via Eritrea 62, 20157 Milan.*

**8th International Conference on Atomic Spectroscopy**
July 1-6, Cambridge, U.K.
*Association of British Spectroscopists, P.O. Box 109, Cambridge CB1 2HY, U.K.*

**2nd Canadian World Chromatography Conference**
July 5-6, Toronto, Canada.
*V. M. Bhatnagar, P.O. Box 1779, Cornwall, Ontario K6H 5V7, Canada.*

**Summer School on Automatic Methods of Analysis**
July 9-13, Swansea, U.K.
*D. Porter, Laboratory of the Government Chemist, Cornwall House, Stamford Street, London SE1 9NQ, U.K.*

**Analysis '79: Automation in Industrial and Clinical Chemistry**
July 16-18, London, U.K.
*Scientific Symposia Ltd., 33-35 Bowling Green Lane, London EC1R 0DA, U.K.*

**3rd International Bioanalytical Forum**
September 4-7, Guildford, U.K.
*Dr. E. Reid, Wolfson Bioanalytical Centre, University of Surrey, Guildford, GU2 5XH, U.K.*

**Automation and Computerisation in the Medical Laboratory**
September 5-7, Stirling University, Scotland.
*G. W. Thomson, Secretary, IMLS Glasgow Branch, 67 Glen Ogilvie, St. Leonards, East Kilbride, Scotland.*

**International Conference on Flow Analysis**
September 11-13, Amsterdam, The Netherlands.
*Secretary FA-Amsterdam, Laboratory for Analytical Chemistry, University of Amsterdam, Nieuwe Achtergracht 166, 1018 WV Amsterdam, The Netherlands.*

**Communications in Microprocessor Industrial Instrumentation**
September 12-13, London, U.K.
*Mrs. P. Keiller, Sira Institute, South Hill, Chislehurst, Kent BR7 5EH, U.K.*

**Le Chromatographe Automatique Industriel en Lague: Analyseur Sophistique ou Capteur Industriel?**
October 3-5, Arles, France.
*Institute de Regulation et Automation Guy Berthier, Chemin des Moines, 13644, Arles, France.*

**Real-time Datahandling and Process Control**
October 23-25, Berlin, West Germany.
*Congress Organisation Company, John Foster Dulles Allee 10, D-1000 Berlin, West Germany.*

### 1980

**Automation at SAC 80**
July 20-26, Lancaster, U.K.
*The Secretary, Analytical Division, The Chemical Society, Burlington House, London W1Y 0BN, U.K.*

### 1981

**Euroanalysis IV**
August 23-28, Helsinki, Finland.
*Association of Finnish Chemical Societies, Executive Secretary, Pohj, Hesperiankatu 3B10, SF-00260 Helsinki 26, Finland.*

**Editor's Note:**
*Organisers of conferences, seminars etc. should send details for inclusion in this calendar as soon as the relevant information is available and not later than three months before the event.*

### Product News

### Laboratory instrumentation and equipment

ChemLab Instruments Ltd., will be exhibiting a selection of items from their wide range of laboratory instrumentation and equipment at Labex '79, 12-16 March 1979. Several new products are to be shown at this exhibition.

A new peristaltic pump with stand-by facility to reduce reagent consumption and a new design of analytical cartridges using AAII type chemistries have been added to the well-established range of equipment for continuous flow analysis. The new Triton 3 micro-computer for use as a general purpose laboratory data processor will also be on display. Amongst the increasing range of analytical programmes is a system for handling simultaneously the results from a number of different gas chromatographs and high performance liquid chromatography.

Other products that will be on display include Nichiryo dispet and micro-dispet dispensers, an improved range of ultrafiltration equipment, Brinkmann probe colorimeters, Wescor osmometers, the Prolabo HPLC system and the W.T.W. Combibox from Germany, which is for checking continuously pH, conductivity and temperature of water effluents.
*ChemLab Instruments Ltd., Hornminster House, 129 Upminster Road, Hornchurch, Essex RM11 3XJ, U.K.*

### Measurement of blood clotting

Blood clotting disorders can now be studied more specifically using a series of methodologies made available recently.

These methodologies use a synthetic chromogenic substrate enabling specific photometric measurement of coagulation chemistries to be done with ease. There are methods for evaluations of antithrombin III, herapin and antiplasmin.

Automation of these tests are specifically suited to the Vitatron PA800, a fully automated programmable clinical analyser. For smaller workloads the Vitatron Reaction Rate Photometer (RRP) is ideal. This instrument when used in conjunction with high precision dispensers and diluters for reagent addition enables accurate measurement of both high and low concentrations of blood clotting factors.
*MSE Scientific Instruments, Manor Royal, Crawley, Sussex RH10 2QQ, U.K.*

### Optical system

The InfraAlyzer 400 is the result of three years research and development and is a natural heir of the earlier InfraAlyzer models from Technicon Industrial Division. The system operates in 8 seconds automatically and incorporates the infra-vision screen which allows commodity recognition. It operates with up to 20 filters instead of the original 6, so allowing a wider range of products to be analysed making it ideal for research into new applications.

The optical system incorporates an integrated sphere detector which provides continuous reference correction and high signal to noise ratios. The essential benefits are accuracy, no drift and faster performance.
*Technicon Instruments Co. Ltd., Evans House, Hamilton Close, Basingstoke, Hants. RG21 2YE, U.K.*

### Microprocessor

Pericom has announced the third model in its range of microprocessor controlled visual display terminals. The 6803 has all the ergonomic features in the 6800 series but allows in addition switching between 80 column and 132 column format. Sophisticated scan electronics have been used allowing the display to be driven to the very edges of the 15" tube. A special character set has been developed for the 132 column format retaining true lower case. The result is a clear, easy to read 132 column format and extra large 80 column format. The 6803 is primarily designed for use with printers and associated software, an area where 80 column VDU format has always been a problem.
*Pericom Data Terminals Ltd., 1/3 Burners Lane, Kiln Farm, Milton Keynes, Bucks., U.K.*

### Automatic sampler

EG&G Princeton Applied Research Corporation has introduced the Model 318 automatic sampler, an interface which permits continuous monitoring of process streams, reaction vessels, and effluents by polarography or stripping voltammetry. The Model 318 interfaces with their Model 174-12 and 374-3 Polarographic analyzers, and automatically provides a signal to trigger external devices such as a recorder or a dispenser.

The sampler consists of an electronics module and a flow-through cell. The electronics module contains a solenoid that is controlled by timing circuitry within a module. The sample is led to the module under positive pressure from gravity, a pump, or pressure. The solenoid opens every 0, 15 or 30 minutes after the preceeding analysis is completed and allows the sample to flow through the cell to completely flush out the old sample. After the sample is changed, the polarographic analyzer automatically performs the analytical determination.
*Princeton Applied Research Corporation, PO Box 2565, Princeton, NJ 08540, U.S.A.*

### Liquid chromatographs

Altex has introduced a range of compact programmable, liquid chromatographs, designed on the principle of distributed intelligence. These units use the Z80 microprocessor to combine easy-to-use programming with full automatic operation.

The Model 330 provides precise, isocratic operation for excellent quantitative and qualitative results. It offers a 6,000 psi constant flow pumping system, with a choice of detectors and a universal sample injector. The total system requires less than 20" bench space.

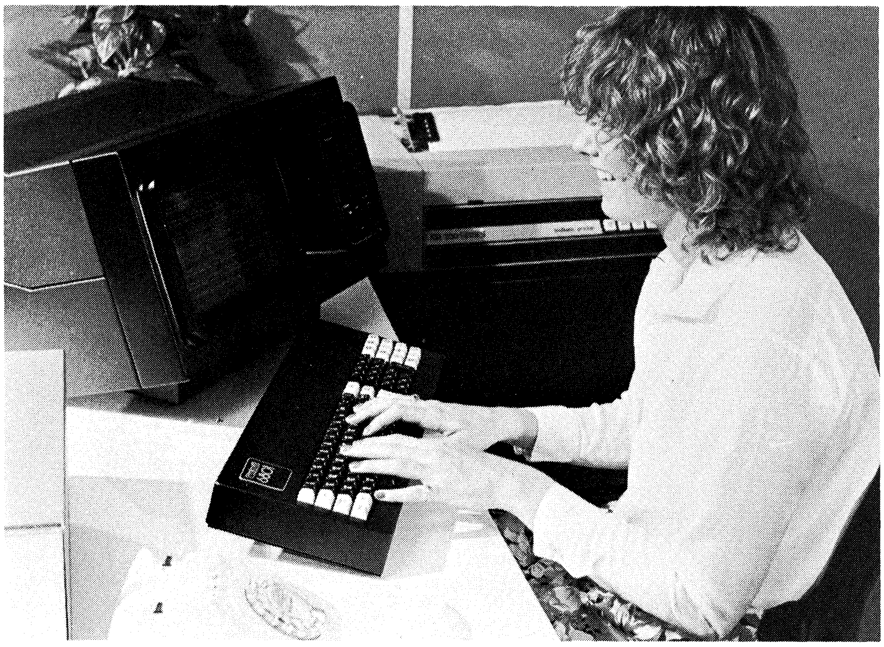

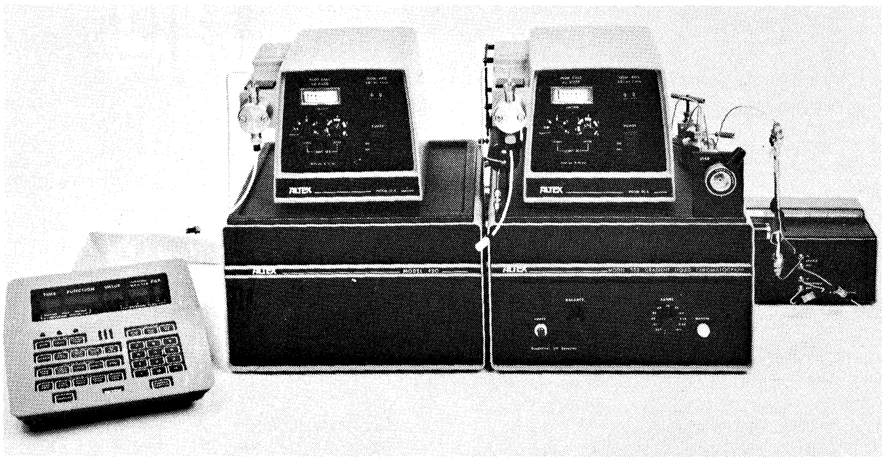

*Top: The 6803 microprocessor controlled visual display terminal from Pericom.*
*Above: The Model 330 liquid chromatograph from Altex.*

The Model 332 is a full research instrument providing an almost infinite number of solvent compositions and flow profiles to tackle difficult separation problems. The microprocessor offers advanced features such as control of solvent reservoir selection, automatic sample injection, column switching and sample collection. Up to 19 different methods can be stored in the memory for recall at any time.
*Altex Scientific Inc., 1780 Fourth Street, Berkeley, California 94710, U.S.A.*

### Process titration

The Model 1800 DigiChem process titrator, manufactured by Ionics is a full automated general purpose analyser specifically designed for on-line monitoring or process control applications. The titrator may be used to determine any inorganic or organic species and dissolved metals for which titration methods are available. Typical applications include pH control, acid and caustic control, scrubber solution monitoring, analysis of chlorine and hypochlorite in bleach and water hardness and alkalinity determinations. It measures the volume of reagent required for the specified endpoint on a measured volume of sample, automatically subtracts a titration blank, calculates the answer in the specified engineering units and displays or transmits the result on the selected output mode. The instrument can be further programmed to treat double endpoint data arithmetically and obtain the desired results directly without manual calculation.

An important design feature is that, unlike conventional titrators this model is capable of measuring two endpoints during each cycle. Endpoints are determined by monitoring the first derivative of the potentiometric titration curve, thus giving high accuracy and eliminating the need for electrode calibration.
*Techmation Ltd., 58 Edgware Way, Edgware, Middx. HA8 8JP., U.K.*

### Automatic injector

Micromeritics Instruments Corporation has announced the Model 725 Automatic Injector for use with high performance liquid chromatography systems. This allows automatic, unattended operation for injection of up to 64 consecutive samples with negligible carry-over between samples.

The injector is microprocessor operated to control the injections per sample vial, injection time (1-99 minutes) rinse between samples and automatic shut down upon completion of the last sample or detection of a malfunction. It uses disposable glass sample vials and the vial cap functions as a piston to discharge the sample with positive flow (not suction or pneumatic). The motorized injection valve operates to 6,000 psi and a 10 up loop is standard.

The injector can be used with any high performance liquid chromatography system. It is designed to accept external control by a computer or computing integrator.

*Micromeritics Instrument Corporation, 5680 Goshen Springs Road, Norcross, Georgia 30093, U.S.A.*

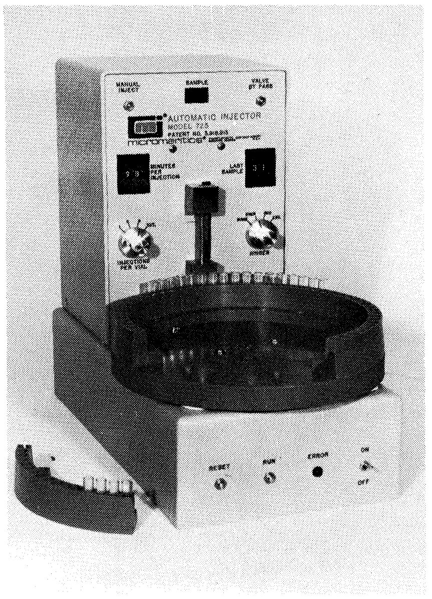

*The Model 725 Automatic Injector from Micrometrics Instruments.*

### Microprocessor programmer

Tenney Engineering Inc., have standardised several previously optional performance features on their DigiTenn microprocessor programmer. The programmer is a digital display, push-button microprocessor programmer that automatically signals environmental test chamber controls to perform time-related temperature, humidity and altitude programs as a one-parameter test or to perform any two parameters simultaneously. It replaces cams and punched tape devices and offers a reliable method of programming functional sequences in a test chamber. The standardised features include 51 program segments that permit a lengthy, non-repetitive program cycle with programming up to 51 steps, seven event switches, furnished with one basic relay, that offer time-related switch closures, a battery back-up system that retains program memory up to 90 minutes during a power loss and compatibility with teletype tape or other ASCII input. In addition the programmer now also offers a standard power on/program erase switch that permits the total shutdown and optional compatibility with IEEE bus.

*Tenney Engineering Inc., 1090C Springfield Road, Union, NJ 07083, U.S.A.*

### Clinical analyser for serum iron and TIBC

The ESA Ferrochem serum iron/TIBC analyser is now available from MSE. This utilises a unique electrochemical process. Calibration and operation is fast and simple, so that single or multiple samples can be run as required any time during day or night. Analysis time for serum iron is 60 seconds, TIBC preparation requires a five minute ion exchange, non-centrifugation step as compared with the 40 or more minutes required by other methods. 10 µl of serum are required for the serum ion determination and 50 µl for TIBC. The instrument is simple to operate, with auto-blank facilities and no preparative chemistry.

*MSE Scientific Instruments, Manor Royal, Crawley, West Sussex RH10 2QQ, U.K.*

### Aerosol and particulate monitoring

Phoenix Precision Instruments have announced a new range of aerosol systems for detecting and monitoring airborne contaminants. The systems comprise Models JM-5000 featuring logarithmic output; JM-6000 with range selectable linear amplification and JM-7000 a combination log/linear instrument. Designed for testing and monitoring HEPA filters, these photometers feature a high sampling rate 100% solid state electronics, and a choice of 5-decade logarithmic, 5-range linear or combined display.

*Techmation Ltd., 58 Edgware Way, Edgware, Middx., U.K.*

### Sample injector

Altex have introduced a sample injection device for high performance liquid chromatography, based upon a four-port valve design rather than the conventional six-port. Constructed in 316 SS and new chemically inert polymers, it is designed for operation at pressures up to 10,000 psi. The design provides easy access to all fittings and requires very little force to actuate from the load to inject positions. Sample loops from 5 to 2,000 up are available, and smaller samples can be injected by partially filling the loop with a standard chromatographic syringe. Available options include pneumatic actuation and an event marker.

*Altex Scientific Inc., 1780 Fourth Street, Berkeley, California 94710, U.S.A.*

### Data acquisition system

Burr-Brown have followed the introduction of two self-contained hybrid 12-bit resolution data acquisition systems with a third — the SDM 858 data acquisition system — which is expressly designed for use with transducers with very low outputs.

The SDM 858 provides eight differential or sixteen single-ended inputs. It contains a high performance instrumentation amplifier, a 16-way CMOS analogue multiplexer, a 12-bit digital-to-analogue converter, voltage reference, channel address counter, and a three-state output buffer stage. The inputs and outputs of the various stages are brought out to package pins, thereby providing the designer with a great deal of flexibility in the way the unit is configured. It operates from ±15 V and 5 V supplies over the temperature range 0 to 70°C and is housed in a metal package designed for printed circuit board mounting measuring 116.8 x 76.2 x 9.5 mm.

*Burr-Brown International Ltd., 17 Exchange Road, Watford, Herts. WD1 7EB, U.K.*

### Clinical analyser

The first in a series of new products to be introduced by LKB Clinicon Systems Ltd., into the United Kingdom market is a compact microprocessor controlled clinical analyser, the ChemCode.

The analyser has been designed to provide speed, simplicity and versatility of operation, which with a suitable reagent programme will perform both enzyme and substrate analyses. It is pre-programmed to perform 20 different tests by fixed time, end point of constant rate methods, and as such is suited to application in laboratories with greatly varied workloads. It can be used to complement both the multichannel analysers and batch analysers. Because of the speed of operation it is suited for emergency sample analysis and on-call analytical demands. The additional security of a hard copy of results is provided to help eliminate possible transcription errors.

*LKB Clinicon Systems Ltd., Bell Lane, Lewes, East Sussex BN7 1LG, U.K.*

